# An integrative U method for joint analysis of multi-level omic data

**DOI:** 10.1186/s12863-019-0742-z

**Published:** 2019-04-10

**Authors:** Pei Geng, Xiaoran Tong, Qing Lu

**Affiliations:** 1 Department of Mathematics, Illinois State University, Normal, IL, 61761 USA; 2 Department of Epidemiology and Biostatistics, Michigan State University, East Lansing, MI, 48824 USA

**Keywords:** Non-parametric method, Functional data analysis, Integrative analysis

## Abstract

**Background:**

The advance of high-throughput technologies has made it cost-effective to collect diverse types of omic data in large-scale clinical and biological studies. While the collection of the vast amounts of multi-level omic data from these studies provides a great opportunity for genetic research, the high dimensionality of omic data and complex relationships among multi-level omic data bring tremendous analytic challenges.

**Results:**

To address these challenges, we develop an integrative U (IU) method for the design and analysis of multi-level omic data. While non-parametric methods make less model assumptions and are flexible for analyzing different types of phenotypes and omic data, they have been less developed for association analysis of omic data. The IU method is a nonparametric method that can accommodate various types of omic and phenotype data, and consider interactive relationship among different levels of omic data. Through simulations and a real data application, we compare the IU test with commonly used variance component tests.

**Conclusions:**

Results show that the proposed test attains more robust type I error performance and higher empirical power than variance component tests under various types of phenotypes and different underlying interaction effects.

**Electronic supplementary material:**

The online version of this article (10.1186/s12863-019-0742-z) contains supplementary material, which is available to authorized users.

## Background

With rapidly evolving high-throughput technologies and ever-decreasing costs, it has become feasible to systematically study diverse types of omic data in biological and clinical studies [1, 2]. The collection of multi-level omic data from these studies provides us a great opportunity to integrate information from different levels of omic data into association analysis [3–6]. Although omic-based association analysis holds great promise for discovering novel disease-associated biomarkers, the discovery process is hampered by the lack of appropriate statistical tools to consolidate and analyze multi-level omic data. The development of advanced statistical methods to address the analytical challenges faced by ongoing omic data analysis can enhance our ability to identify new disease-associated biomarkers.

Comprehensive reviews of integrative analysis on multi-level omic data are summarized in [3, 5, 7] and the references therein. Most of the existing methods for integrative analysis are developed based on score-type tests or variance component tests. For instance, in the integrative analysis of single-nucleotide variants (SNVs) and transcript expression data, [6] used the estimating equations to estimate parameters of interest, and then proposed a Wald test to evaluate the association between the outcome and a set of genetic variants, considering possible interactions. In order to efficiently test the joint effects of SNVs and gene expression with a binary phenotype, [8] developed a combined variance component test in the mixed model framework. Based on this work, [9] further investigated a variance component score test for modeling multiple genomic data including SNVs, gene expression, and methylation data, each of which can come from different samples or studies. While those methods have attractive properties under various scenarios, most of these methods are parametric-based or semi-parametric-based, which often rely on a distribution assumption (e.g., a normal distribution assumption). When this assumption is violated, these methods are subject to false positive results and/or power loss [10]. The diagnostic assessments of human diseases can often be of different types (e.g., binary, ordinal and continuous) and follow known or unknown distributions. This issue is, however, paid less attention by the existing methods.

Moreover, the molecular complexity of human diseases manifests itself at the genomic, transcriptomic, epigenomic and proteomic levels [11, 12]. Different levels of omic data can interact in the disease process. By considering interactions between different levels of omic data, the power of detecting disease-associated biomarkers can be potentially enhanced. While some of existing methods consider interactions between omic data [6, 8], they commonly assume a particular interaction model (e.g., a multiplicative model), and are subject to suboptimal performance if the underlying model has different forms (e.g., a threshold model).

To address these limitations, we propose a non-parametric framework for association analysis using multi-level omic data. The IU test is a U-statistic-based test, which is constructed using the pairwise omic and phenotype similarities of subjects. It has several remarkable features worthy of attention: 1) it makes no distribution assumptions, and therefore provides a robust and powerful performance when analyzing phenotypes and omic data with unknown distributions; 2) it provides a unified framework for analyzing various types of phenotypes and omic data (binary, ordinal and continuous); and 3) it considers interactions among different levels of omic data without posing specific model assumptions.

The remaining of the paper is organized as follows. We begin with a detailed description of the proposed integrative U method in “Methods” section, and then present the simulation results of the IU method under different types of phenotypes and various genetic or interaction effects in “Simulation” section. Using the proposed method, we performed an integrative analysis of the DNA sequencing and gene expression data from a hypertension study in “An integrative analysis of gene and gene expression data of hypertension” section. “Conclusion” section summarizes the advantages and limitations of the IU test. Details of the proof of the main results can be found in the Additional file 1.

## Methods

Suppose that we are interested in evaluating the joint association of M levels of omic data with a disease phenotype of interest. Without loss of generality, we illustrate the method with two levels of omic data (i.e., SNVs and gene expression data). The extension to more than 2 levels of omic data will be discussed later in “Conclusion” section. Let *Y*_*i*_ be a continuous or discrete disease phenotype, *S*_*i*_ be a scalar gene expression variable, and *G*_*i*_=(*G*_*i*_(*t*_1_),*G*_*i*_(*t*_2_),...,*G*_*i*_(*t*_*p*_)) be the genotypes of *p* SNVs (e.g., coding variants in a gene) for the *i*th individual (*i*=1,......,*n*), where *t*_*j*_ is the SNV location and *G*_*i*_(*t*_*j*_)=0,1,2 is coded as the number of minor alleles.

### Genetic smoothing

In recent literature, functional data analysis has been often applied to handle the genetic data. For instance, [13] proposed a functional linear model for quantitative traits using B-spline basis functions to expand the genotype functions. Vsevolozhskaya et al. [14] proposed a functional analysis of variance method to test the association of sequence variants in a genomic region with a qualitative trait. Functional data analysis has also been developed for different types of traits and study purposes in genetic research. For instance, [15] developed a Cox proportional hazard model with functional regression for gene-based association analysis of survival traits. Moreover, [16] proposed a generalized functional linear model to perform meta-analysis of multiple studies to evaluate the association of genetic variants with dichotomous traits.

**Fig. 1:**
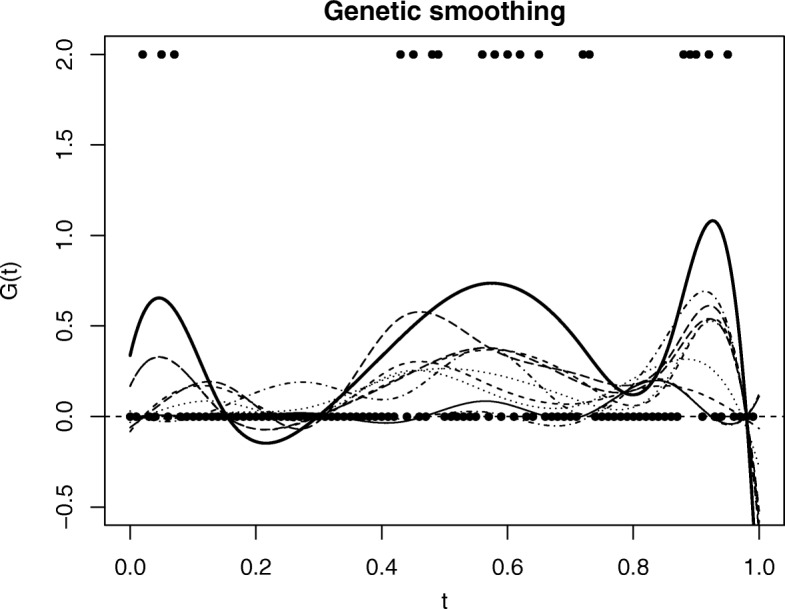
A simple illustration of fitted B-spline curves for genotype sequence. The solid dots present the genotype sequence for one subject, and the solid thick curve is the corresponding smooth curve. In total, a sample of 10 subjects are simulated and the 10 corresponding smoothed curves are presented

Here we adopt the functional data analysis to handle the SNVs. Rather than assuming *G*_*i*_(*t*_*j*_) as a random variable, we assume that *G*_*i*_(*t*_*j*_) is a discrete realization of a function *G*_*i*_(*t*) generated from a stochastic process with mean function *η*(*t*) and covariance function *Γ*(*s*,*t*). The B-spine smoothing technique is then used to model the underlying function curve *G*_*i*_(*t*). In other words, *G*_*i*_(*t*) can be written as a linear sum of specified basis functions:

$$G_{i}(t) = \sum\limits_{k=1}^{K} \beta_{ik} B_{k}(t), $$

where {*β*_*k*_(*t*),*k*=1,...,*K*} is the polynomial basis functions in *L*_2_ Hilbert space. The fitted smoothing curves are demonstrated in Fig. 1. Similar to other functional-based methods [14], we implement the smoothing by scaling the locations to the interval [0,1], and use the penalization technique to determine the appropriate number of knots (i.e., smoothness).

### Test statistic

With the assumptions of *Y*, *G*(*t*) and *S* mentioned above, we aim to test the hypotheses:

*H*_0_: *Y* is independent of *G*(*t*) and *S*;

*H*_*a*_: *Y* is associated with *G*(*t*) or *S*.

Since we do not assume any regression form of the association between *Y* and the genetic variables (*G* and *S*), to perform the hypothesis testing, we propose a non-parametric integrative U statistic defined as

$$\begin{array}{@{}rcl@{}} U_{n} = \frac{1}{n(n-1)}\sum\limits_{i,j=1,i\neq j}^{n} K_{1}(Y_{i}, Y_{j}) K_{2}(S_{i}, S_{j}) \int_{0}^{1} G_{i} (t) G_{j} (t) dt,  \end{array} $$

where *K*_1_(·,·) and *K*_2_(·,·) are symmetric kernel matrices that measure the similarities of two individuals’ phenotypes and gene expression values, respectively. For simplicity, we use the cross product for both kernel matrices

$$\begin{array}{@{}rcl@{}} U_{n} = \frac{1}{n(n-1)}\sum\limits_{i,j=1,i\neq j}^{n} Y_{i} Y_{j} S_{i} S_{j} \int_{0}^{1} G_{i} (t) G_{j} (t) dt.  \end{array} $$

In addition to the cross product kernel, other kernels, such as those proposed in [10] and [17] can also be used.

From the above equation, the proposed test statistic is a U statistic defined on all possible pairs of subjects (*i*,*j*), where the genetic similarity of subjects *i* and *j* is defined as the inner product of the smooth curves of the stochastic process, i.e., $\int _{0}^{1} G_{i} (t) G_{j} (t) dt$. The phenotype similarity and gene expression similarity between the subjects *i* and *j* are simply products of two subjects’ phenotype and gene expression values, respectively.

### Asymptotic property

Under the null hypothesis and the assumption *G*_*i*_(*t*)∼*SP*(*η*(*t*),*Γ*(*s*,*t*)), we obtain

$$\begin{array}{@{}rcl@{}} r_{i} = E(U_{n}|Z_{i}) = \mu_{Y} \mu_{S} Y_{i} S_{i} \int_{0}^{1} G_{i}(t) \eta(t) dt,\\ \quad \mu_{0} = E(U_{n}) = \mu_{Y}^{2} \mu_{S}^{2} ||\eta(t)||^{2}, \end{array} $$

where *Z*_*i*_=(*Y*_*i*_,*S*_*i*_,*G*_*i*_), *μ*_*Y*_ and *μ*_*S*_ are the population means of *Y* and *S*, respectively. The asymptotic result of non-degenerated U statistics in [18] implies that

$$\sqrt{n}(U_{n} - \mu_{0}) \rightarrow N\left(0, 4\sigma_{1}^{2}\right), $$

where $\sigma _{1}^{2} = Var(r_{1})$. Moreover, by applying the result of stochastic processes in Section 4.2 of [19], we can further obtain that

$$\begin{array}{@{}rcl@{}} Var(r_{1}) & = & \mu_{Y}^{2} \mu_{S}^{2} Var\left(Y_{1} S_{1} \int G_{1}(t)\eta (t) dt\right)\\ & = & \mu_{Y}^{2} \mu_{S}^{2} \left(\sum\limits_{k=1}^{m} \lambda_{k} \delta_{k}^{2}\right) \left(\mu_{Y}^{2} + \sigma_{Y}^{2}\right)\left(\mu_{S}^{2} + \sigma_{S}^{2}\right)\\ &&+\mu_{Y}^{2} \mu_{S}^{2} \left(\mu_{Y}^{2} \sigma_{S}^{2} + \mu_{S}^{2} \sigma_{Y}^{2} + \sigma_{Y}^{2} \sigma_{S}^{2}\right) ||\eta ||^{4}, \end{array} $$

where *m* is the number of eigenvalues of *Γ*, (*λ*_*k*_,*ϕ*_*k*_(*t*)) are eigenvalues and eigenfunctions of the covariance function *Γ*, $\delta _{k} = \int \phi _{k}(t) \eta (t) dt$, $\sigma _{Y}^{2}$ and $\sigma _{S}^{2}$ are the population variances of *Y* and *S*.

Because *μ*_0_ is unobservable, we propose to estimate it by substituting the population means of *Y*, *S* and *G*(*t*) by their corresponding sample means, i.e.,

1 $$\begin{array}{@{}rcl@{}} \hat\mu_{0} = \bar{Y}^{2} \bar{S}^{2} ||\bar{G}(t)||^2. \end{array} $$

Let $s_{Y}^{2}$ and $s_{S}^{2}$ be the sample variances of *Y* and *S*, $\hat \lambda _{k}$ and $\hat \phi _{k}$ be the eigenvalues and eigenfunctions of $\hat \Gamma (s,t) = \frac {1}{n}\sum _{i=1}^{n} \left (G_{i}(t)-\bar {G}(t)\right)\left (G_{i}(s) - \bar {G}(s)\right)$. By letting $\hat \delta _{k} = \int _{0}^{1} \hat \phi _{k}(t)\bar G(t)dt$, we can obtain the asymptotic distribution of the test statistic under *H*_0_:

#### **Theorem 1.**

*Assume that both Y and S have finite means and variances and* *G*(*t*)∼*SP*(*η*(*t*),*Γ*(*s*,*t*)). *Moreover,* *μ*_*Y*_*μ*_*S*_≠0*and S is independent of* *G*(*t*). *Then, under* *H*_0_,

$$T_{n} = \frac{\sqrt{n}(U_{n} - \hat\mu_{0})}{\hat{\sigma}}\to N(0,1), $$

*where*$\hat \mu _{0}$*is defined in* (1) *and*$\hat \sigma ^{2} = 4\sum \hat \lambda _{k} \hat \delta _{k}^{2} \left \{\bar Y^{2} \bar S^{2} \left (\bar Y^{2} s_{S}^{2} + \bar S^{2} s_{Y}^{2} + s_{Y}^{2} s_{S}^{2}\right)\right \} + 4\bar Y^{2} \bar S^{2} ||\bar G||^{4} s_{Y}^{2} s_{S}^{2}.$

We would reject *H*_0_ if $|{\sqrt {n}(U_{n} - \hat \mu _{0})}/{\hat \sigma }|>z_{\alpha /2}$, where *z*_*α*/2_ is the upper *α*/2 quantile of the standard normal distribution.

#### **Remark 1.**

The assumption of the underlying stochastic process is very general. We do not need a specific condition on the pointwise distributions such as Gaussian, which is the required assumption in [14].

#### **Remark 2.**

The proposed test inherits the robustness property from U statistics, and is capable of handling both discrete and continuous phenotypes with various underlying distributions. Moreover, the proposed test does not need to specify any form of the regression function *μ*=*E*(*Y*|*S*,*G*), hence the test procedure is free of model assumptions.

The method can also be used for different study purposes. For instance, to only test the effect of SNVs (e.g., in a genetic association study), the corresponding integrative U test statistic can be simplified as $U_{G} = \frac {1}{n(n-1)}\sum \limits _{i\neq j} Y_{i} Y_{j} \int _{0}^{1} G_{i} (t) G_{j} (t) dt$ with $\hat \mu _{G} = \bar Y^{2} ||\bar {G}(t)||^{2}$ and variance estimator $\hat \sigma _{G}^{2} = 4\hat \mu _{Y}^{2} s^{2}_{Y} \sum \hat \lambda _{k} \hat \delta _{k}^{2} $.

### Power and sample size

While omic-based studies become increasingly popular in human genetic research, few statistical tools are available for power and sample size calculation. In this section, we investigate the power of the proposed method under certain alternative hypotheses and provide a convenient way for power/sample size calculation.

We have studied the IU test under the null hypothesis, $E\left \{Y_{i} Y_{j} S_{i} S_{j} \int _{0}^{1} G_{i}(t) G_{j}(t)dt\right \} = \mu _{0} = \mu _{Y}^{2} \mu _{S}^{2} ||\eta ||^{2}$. Under the alternative hypothesis, we assume that $E\left \{Y_{i} Y_{j} S_{i} S_{j} \int _{0}^{1} G_{i}(t) G_{j}(t)dt\right \}$ =*μ*_1_≠*μ*_0_ and *Var*(*Y*_*i*_*Y*_*j*_*S*_*i*_*S*_*j*_$ \left. \int _{0}^{1} G_{i}(t) G_{j}(t)dt|Y_{i}, S_{i}, G_{i}\right) = \tau _{1}^{2}$. Without loss of generality, we assume that *μ*_1_>*μ*_0_. Applying the Hoeffding projection in [18], we obtain that $\frac {\sqrt {n}(U_{n} - \mu _{1})}{2\tau _{1}}\to N(0,1)$. Hence, under the alternative hypothesis, *U*_*n*_ can be written as

$$\frac{\sqrt{n}(U_{n}-\hat\mu_{0})}{\hat\sigma} = \frac{2\tau_{1}}{\hat\sigma}\frac{\sqrt{n}(U_{n} - \mu_{1})}{2\tau_{1}} + \frac{\sqrt{n}(\mu_{1} - \hat\mu_{0})}{\hat\sigma}. $$

Since both $\hat \mu _{0}$ and $\hat \sigma $ are consistent estimators of *μ*_0_ and *σ*, for a sufficiently large *n*, we have

$$\frac{\sqrt{n}(U_{n}-\hat\mu_{0})}{\hat\sigma} \sim N\left(\frac{\sqrt{n}(\mu_{1} - \mu_{0})}{\sigma}, \frac{4\tau_{1}^{2}}{\sigma^{2}}\right). $$

Therefore, the power of the proposed test can be calculated by

$$\begin{array}{@{}rcl@{}} && P\left(\left|\frac{\sqrt{n}(U_{n} - \hat\mu_{0})}{\hat\sigma}\right|>z_{\alpha/2}\right) \\ & \geq & P\left(\frac{\sqrt{n}(U_{n} - \hat\mu_{0})}{\hat\sigma}>z_{\alpha/2}\right) \\ &\geq & P\left(\frac{\sqrt{n}(U_{n} - \mu_{1})}{2\tau_{1}}> \frac{\hat\sigma}{2\tau_{1}}\left(z_{\alpha/2} - \frac{\sqrt{n}(\mu_{1} - \mu_{0})}{\hat\sigma}\right)\right) \\ & = & \Phi\left(\frac{\hat\sigma}{2\tau_{1}}\left(\frac{\sqrt{n}(\mu_{1} - \mu_{0})}{\hat\sigma}- z_{\alpha/2}\right)\right). \end{array} $$

For desired power *β*, one can derive the minimal required sample size by setting the inequality $\Phi \left (\frac {\sigma }{2\tau _{1}}\left (\frac {\sqrt {n}(\mu _{1} - \mu _{0})}{\sigma }- z_{\alpha /2}\right)\right)\ge \beta $, therefore the required sample size can be calculated by

$$n = \min_{m\in \mathbb{Z}} \left\{m\geq \frac{\sigma^{2}}{(\mu_{1}-\mu_{0})^{2}}\left(z_{\alpha/2} + \frac{2\tau_{1} z_{\beta}}{\sigma}\right)^{2}\right\}. $$

## Results

### Simulation

Through simulations, we compared the type I error and empirical power of the proposed test with those of two variance component methods: the adjusted kernel sequencing association test and variance component test proposed by [8]. Since the original kernel sequencing test developed by [17] is proposed for only sequencing SNVs, we slightly modify the method to incorporate gene expression data in the test. Recall that the sequence kernel association test (SKAT) proposed by [17] has the form

$$Q = (\mathbf{Y}-\hat{\mathbf{\mu}})^{T} \mathbf{K} (\mathbf{Y} - \hat{\mathbf{\mu}}), $$

where **Y**=(*Y*_1_,...,*Y*_*n*_)^*T*^ and $\mathbf K(G_{i},G_{j}) = \sum _{k=1}^{p} w_{k} G_{ik} G_{jk}$. To make the methods comparable, the adjusted SKAT (Adj-SKAT) is modified as:

$$\tilde Q = (\mathbf{Y}-\hat{\mathbf{\mu}})^{T} \tilde{\mathbf{K}}_{S} (\mathbf{Y} - \hat{\mathbf{\mu}}), $$

where the elements of $\tilde {\mathbf {K}}_{S}$ are defined as $ \tilde {\mathbf K}_{S}(G_{i},G_{j}) = \sum _{k=1}^{p} w_{k} S_{i} S_{j} G_{ik} G_{jk}$.

Interestingly, the proposed *U*_*n*_ has a similar form of *Q*. If we define

$$\mathbf K_{S}(G_{i},G_{j}) = S_{i}S_{j}\int_{0}^{1} G_{i}(t) G_{j}(t)dt, $$

and further define metric **K**_*S*_=(*K*_*S*_(*G*_*i*_,*G*_*j*_))_*n*×*n*_ with zeros as diagonal elements, then the proposed U statistic *U*_*n*_ can be written as a similar form to Adj-SKAT:

$$U_{n} = \mathbf{Y}^{T}\mathbf{K}_{S}\mathbf{Y}.$$

In addition to Adj-SKAT, we also compared IU test with the component variance test (VCT) developed under the generalized linear mixed model framework by [8]. VCT is proposed to test the joint effects of SNVs and gene expression, and is defined as

$$\bar Q = \frac{1}{n} (\mathbf{Y}-\hat{\mathbf{\mu}})^{T}\left\{a_{1} \mathbf{G}\mathbf{G}^{T} + a_{2} \mathbf{S}\mathbf{S}^{T} + a_{3} \mathbf{C}\mathbf{C}^{T}\right\}(\mathbf{Y}-\hat{\mathbf{\mu}}),$$

where (*a*_1_,*a*_2_,*a*_3_) are weight parameters and **C** is the product of SNVs and gene expression. In the simulation, we used the recommended weight, the inverse of the square root of the variance, as suggested by [8].

The genetic data was simulated from the 1000 Genome Project [20]. Specifically, we used a 1Mb region of the genome (Chromosome 17: 7344328-8344327) from 1092 individuals in 1000 Genome Project. In each simulation replicate, SNVs were generated by randomly choosing a segment with *p*=100 consecutive SNVs from the genome. Then the stochastic smoothing function curves were constructed by applying the functional data analysis to the SNV sequences. Gene expression data was generated from a normal distribution, *N*(1,1.2^2^). The natural cubic spline smoothing with penalty parameter introduced in [14] was applied. All the results of type I error and empirical power were calculated based on 1000 simulated replicates.

#### Type I error performance

The phenotype assessments can often be of different types (e.g., binary and continuous phenotypes), with unknown underlying distributions. In this simulation, we evaluated the robustness of three methods against different phenotype distributions. Totally, four types of distributions: Bernoulli (binary), Gaussian, T and Double-exponential (DE), were considered in this simulation. Both T and DE have heavier tails than Gaussian. The original VCT test is developed for binary phenotype, but can be extended for other types of phenotypes by using different link functions. For instance, by using the identity function, VCT can be used to analyze continuous phenotypes. Under *H*_0_, *Y* is independently generated from Bernoulli(1/(1+*e*^0.2^)), *N*(1,1), *T*_2_, *T*_4_ and *DE*(1), respectively. Table 1 summarizes the type I error performance of three methods. From Table 1, we find that type I error rates of both VCT and Adj-SKAT are well controlled for Bernoulli- and Gaussian-type of phenotypes, but are inflated under the heavy tailed distributions(T and DE). As we expect from the U statistic property, the IU test attains robust performance under all phenotype distributions.

**Table 1:** Type I error comparison of three methods for different types of phenotypes

phenotype distributions	Bernoulli	Gaussian	*T*_2_	*T*_4_	DE
IU	0.048	0.049	0.052	0.055	0.052
VCT	0.046	0.053	0.091	0.068	0.075
Adj-SKAT	0.035	0.055	0.129	0.062	0.064

Bernoulli, Gaussian, T, and DE correspond to Binary, Gaussian-distributed, T-distributed, and Double-exponential distributed phenotypes. The nominal size of the test is 0.05 and *n*=200

We further investigated the empirical levels of the proposed test for different sample sizes. For this simulation, we considered both continuous and binary phenotypes and varied the study sample size from 100 to 500. A normal distribution was used to simulate the continuous phenotype, while balanced samples were generated for the binary phenotype. 1000 independent simulation replicates were used to obtain the empirical sizes. The empirical levels of the test for nominal sizes 0.05 and 0.01 are summarized in Table 2. As observed in Table 2, the type I error rates of the proposed test are well controlled for both types of phenotypes and different sample sizes.

**Table 2:** Type I error of IU for different sample sizes with nominal sizes 0.05 and 0.01

Type I error with Gaussian phenotype					
*α* / *n*	100	200	300	400	500
0.05	0.049	0.051	0.048	0.047	0.052
0.01	0.01	0.011	0.009	0.011	0.010
Type I error with binary phenotype					
*α* / *n*	100	200	300	400	500
0.05	0.044	0.046	0.048	0.052	0.048
0.01	0.011	0.012	0.009	0.011	0.012

#### Power performance

For the power comparison, we considered the scenarios with or without an interaction between SNVs and gene expression. For the scenarios with an interaction, we studied the performance of the three methods under various interaction models. Similar to the type I error simulation, the genetic data was obtained from the 1000 Genome Project and gene expression *S*_*i*_ was sampled from *N*(1,1.2^2^). The binary response *Y*_*i*_ was then generated from a logistic regression model. In each simulation, we randomly chose 100 cases and 100 controls to form a balanced case-control sample. For continuous phenotypes, we simulated both Gaussian-distributed and T-distributed phenotypes.

**Case 1: No interaction effect**

We first evaluated the power of three methods under the scenario when there is no interaction between SNVs and gene expression. In the binary case, the underlying model is similar to the one assumed in [8]

2 $$\begin{array}{@{}rcl@{}} \text{ Model 1 (a):} logit\{P(Y_{i} =1 | G_{i}, S_{i})\} \\= - 0.2 + G_{i}^{T} \beta_{G} + S_{i} \beta_{S} + S_{i} G_{i}^{T} \gamma, \end{array} $$

where *β*_*S*_ is a scalar parameter, *β*_*G*_ and *γ* are *p*×1 vector parameters. In the continuous case, a linear mixed model was used,

$$\begin{array}{@{}rcl@{}} &\text{ Model 1 (b):} Y_{i} = 2 + G_{i}^{T} \beta_{G} + S_{i} \beta_{S} + S_{i} G_{i}^{T} \gamma + \varepsilon_{i}, \quad\quad \quad \quad \quad\quad \\ &\text{ Model 1 (c):} Y_{i} = 2 + G_{i}^{T} \beta_{G} + S_{i} \beta_{S} + S_{i} G_{i}^{T} \gamma + e_{i}, \quad \quad \quad \quad \quad\quad \end{array} $$

where *β*_*G*_ and *β*_*S*_ were defined as in (2), *ε*_*i*_∼*N*(0,1) and *e*_*i*_∼*T*(2), a T-distribution with 2 degrees of freedom.

Similar to [8], we assume that *β*_*G*_ and *γ* are randomly generated from probability distributions with mean 0 and variances $\sigma _{G}^{2}$ and $\sigma _{\gamma }^{2}$. In this simulation, the genetic effects measured by *β*_*G*_ were generated from a normal distribution, $N(0,\sigma _{G}^{2})$, while the interaction effects measured by *γ* were all set to be zero ($\sigma _{\gamma }^{2} = 0$) in order to study the marginal effects of genetic variables.

**Fig. 2:**
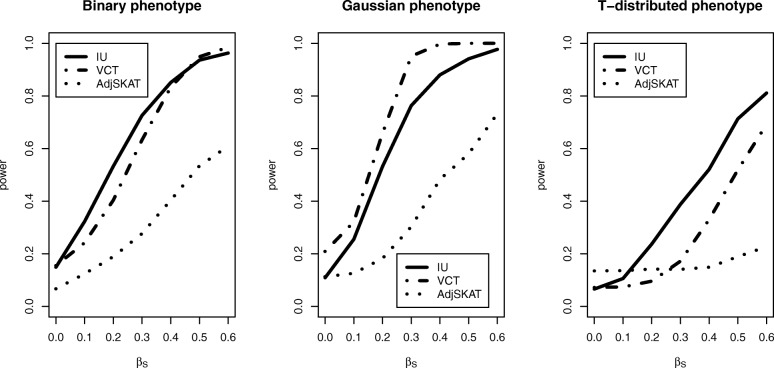
Power comparison of three methods for different types of phenotypes when there is no interaction. *β*_*S*_ stands for the causal effect of gene expression *S*

The power performance for the binary, Gaussian-distributed, and T-distributed phenotypes under the Model 1 is summarized in Fig. 2. The figure shows the power performance of three methods when the effect of gene expression, *β*_*s*_, increases and the effects of SNVs remain the same (*σ*_*G*_=0.1). As shown in the figure, both IU and VCT have higher power than Adj-SKAT as the effect of gene expression increases for binary phenotype. As expected, VCT achieves highest power for Gaussian-distributed phenotype among the three methods. For the T-distributed phenotype, IU outperforms VCT and Adj-SKAT while Adj-SKAT has little power for T-distributed phenotype. Overall, IU test is more robust than the other two methods to the phenotype distributions.

**Case 2: Interaction effect**

We then compared the performance of three methods under a more complex scenario when there is an interaction between SNVs and gene expression. In this simulation, we considered three types of interaction effects: multiplicative, threshold, and random interaction effects. Similar to the simulation with no interaction, we evaluated the methods under three different kinds of phenotypes.

**Fig. 3:**
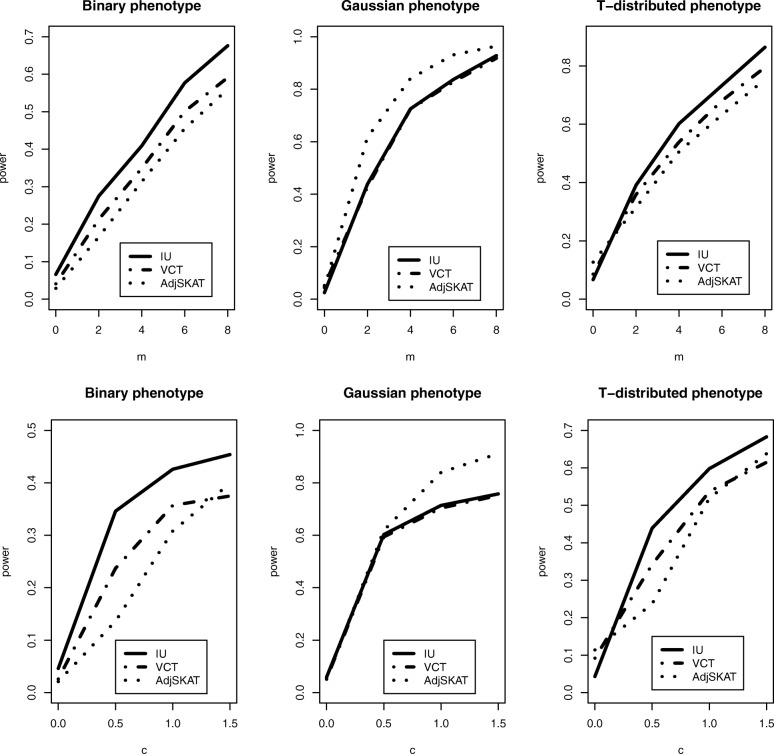
Power comparison of three methods for different types of phenotypes when there is a multiplicative interaction. *m* stands for the number of causal SNVs and *c* stands for the scale of interaction effect in the Model 2

A multiplicative interaction effect is simulated for binary, Gaussian-distributed and T-distributed phenotypes based on the model below,

$$\begin{array}{@{}rcl@{}} && \text{ Model 2 (a):} logit\{P(Y_{i} =1 | G_{i}, S_{i})\} = - 0.2 + c \, S_{i} G_{i}^{T} W, \\ && \text{ Model 2 (b):} Y_{i} = 2 + c \, S_{i} G_{i}^{T} W + \varepsilon_{i}, \\ && \text{ Model 2 (c):} Y_{i} = 2 + c \, S_{i} G_{i}^{T} W + e_{i}, \hspace{1.3in} \end{array} $$

where *c* is a scale parameter, *ε*_*i*_∼*N*(0,1), and *e*_*i*_∼*T*(2). With *W* being a *p*×1 vector, each element of *W* equals to 1 if the corresponding genetic variant is a causal SNV, and 0 if the genetic variant is non-causal. Under the null hypothesis of no association, all elements in W are 0. Let *m* be the number of causal SNVs, then a larger *m* indicates increasing interaction effects on the phenotype. Similarly, a large *c* corresponds to a strong interaction effect. The upper panel of Fig. 3 shows the power comparison of three methods as *m* increases from 0 to 8 with scale parameter *c*=1, and the lower panel of Fig. 3 shows power performance as the scale parameter *c* increases from 0 to 1.5 with *m*=4. As we observe from the figure, IU test attains higher power than VCT and Adj-SKAT for binary and T-distributed phenotypes with the increasing interaction effect. For Gaussian-distributed phenotype, Adj-SKAT has highest power among the three methods because the cross product kernel used in Adj-SKAT perfectly captures the true underlying interaction model.

**Fig. 4:**
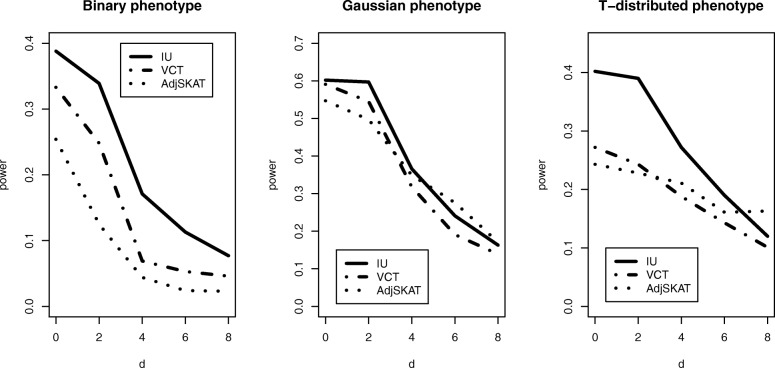
Power comparison of three methods for different types of phenotypes when there is a threshold interaction. *d* is the threshold parameter in Model 3

Next we considered a threshold interaction model, which has the following form:

$$\begin{array}{@{}rcl@{}} && \text{ Model 3 (a):} logit\{P(Y_{i} =1 | G_{i}, S_{i})\}\\ &&= \left\{\begin{array}{ll} - 0.2 + S_{i} G_{i}^{T} W,& \text{if}~ S_{i} G_{i}^{T} W > d,\\ -0.2 &\text{ otherwise, } \end{array} \right.\\ && \text{ Model 3 (b):} Y_{i} = \left\{\begin{array}{ll} 2 + S_{i} G_{i}^{T} W + \varepsilon_{i}, & \text{ if}~ S_{i} G_{i}^{T} W > d,\\ 2 + \varepsilon_{i} &\text{ otherwise, } \end{array} \right.\\ && \text{ Model 3 (c):} Y_{i} = \left\{\begin{array}{ll} 2 + S_{i} G_{i}^{T} W + e_{i}, & \text{ if}~ S_{i} G_{i}^{T} W > d,\\ 2 + e_{i} &\text{ otherwise, } \end{array} \right.\\ \end{array} $$

where *d* is a threshold parameter, *ε*_*i*_ and *e*_*i*_ are the same as those in Model 2. A large *d* corresponds to a weak interaction effect. Under this model, the effect substantially increases when the product of SNV and gene expression exceeds the threshold *d*. Figure 4 shows that all the three tests have decreased power as the threshold parameter *d* increases from 0 to 8. Moreover, the IU test achieves much higher power than the other two methods for the binary and T-distributed phenotypes while it has a similar performance as the other two methods for the Gaussian-distributed phenotype.

**Fig. 5:**
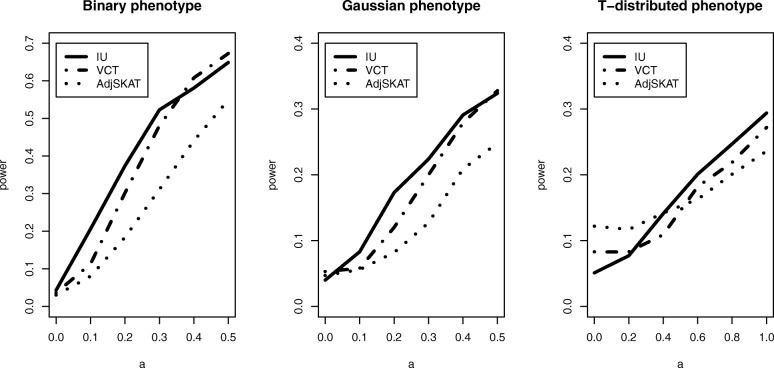
Power comparison of three methods for different types of phenotypes when there is a random effect interaction in Model 1. *a* is the boundary of the uniform distribution used to generate the random effect

**Fig. 6:**
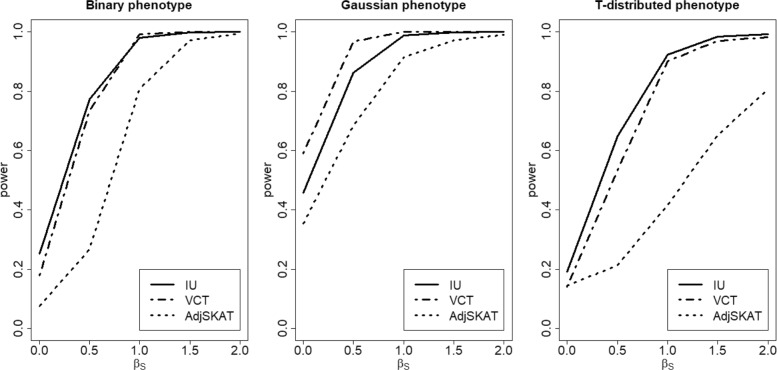
Power comparison of three methods for different types of phenotypes when there are both marginal effects (*σ*_*G*_=0.1, *β*_*S*_=0,0.5,1,1.5 and 2) and random interaction effect (*γ*∼*U*(−0.1,0.1)) in Model 1

Finally, we considered a random effect interaction model based on the model 1 described in (2) with elements of *γ* generated from a uniform distribution *U*(−*a*,*a*),*a*>0. With no marginal effects (i.e., *σ*_*G*_=0, *β*_*S*_=0), Fig. 5 shows that IU test has a similar power performance as VCT for both binary and Gaussian-distributed phenotype. We also find that for the heavy-tailed phenotype (i.e., the T-distributed phenotype), IU attains more robust type I error and higher power than VCT and Adj-SKAT. With both marginal (*σ*_*G*_=0.1, *β*_*S*_=0,0.5,1,1.5,2) and interaction effects (*a*=0.1), the power performance shown in Fig. 6 is similar to that of Case 1 shown in Fig. 2.

In summary, the proposed IU test obtains higher power as the marginal or interaction effects increase. Unlike VCT or Adj-SKAT, which show higher power only under some specific models (e.g., the random effect or cross-product interaction models), the IU test showed more robust and stable performance for different phenotypes and various underlying models. These features make IU more appropriate to use when we have limited knowledge on the actual underlying model.

### An integrative analysis of gene and gene expression data of hypertension

Hypertension is one of the most common chronic diseases, which affects a large proportion of human population worldwide. Despite decades of research in hypertension, the genetic etiology of hypertension remains largely unknown. The successful identification of genetic variants predisposing to hypertension holds promise for providing better understanding of genetic etiology of hypertension and promoting new therapeutic targets. In this application, we performed an integrative analysis of DNA sequencing and gene expression data from the San Antonio Family Heart Study (SAFHS) and the San Antonio Family Diabetes/Gallbladder Study (SAFDGS). SAFHS and SAFDGS include standardized diagnostic assessments of hypertension (i.e., Case vs. Control). Whole-genome sequencing (WGS) data were available on the odd numbered autosomes. In addition, gene expression was measured using peripheral blood mononuclear cells collected at the first examination. In total, there are 260 subjects with WGS data, gene expression data, and the binary hypertension (HTN) phenotype measured.

Prior to the integrative analysis, we performed a quality control and data preparation process. In this process, we assembled multiple SNVs into genes based on the Genome Reference Consortium release version 38 (GRCh38) and excluded genes without gene expression data. To deal with missing values in the genetic data, we imputed the genotype values from multinomial distribution using the sample proportions as the generating probabilities. After data processing step, 2389 genes and the corresponding gene expression remained for the integrative analysis. We then applied a generalized mixed model to the binary HTN phenotype with covariates AGE, MEDS, SMOKE, SEX and the kinship matrix to remove potential confounding effects and the familial correlations. The residuals were used as the responses in this integrative analysis. Eventually, the proposed IU test is applied to detect the joint effect of genes and gene expression data.

**Fig. 7:**
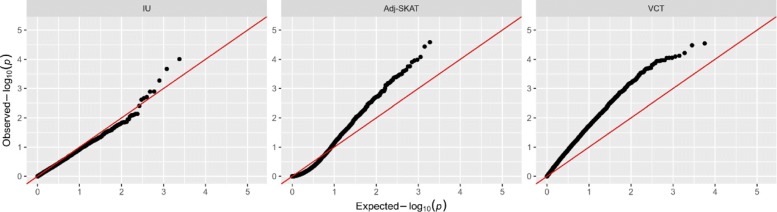
QQplot of the hypertension study using the proposed IU test, Adj-SKAT and VCT

From the Q-Q plot of HTN in Fig. 7, we find no evidence of systematical inflation of the association result. While there is no significant findings after adjusting for multiple testing, there are a few genes reached marginal significance (e.g., *UBAC1*). Among the top findings, some genes may have biological plausibility related to hypertension. For instance, *MFGE8* has been previously reported to up-regulate the intake of Dietary Fats [21], and the Dietary Fats regulates blood pressure via Central Leptin mediated pathways [22]. Therefore, *MFGE8* could be a potential risk factor for hypertension [23]. The expression of *IFI44L* is demonstrably increased upon contact of Nickel [24] or Nickel Chloride [25], which is associated with elevated prevalence of hypertension [26]. Although previous studies suggested that some genes in Table 3 may play a role in hypertension, further studies and biological experiments are needed to confirm the association and to further investigate the potential role of these genes in hypertension.

**Table 3:** Top 10 gene findings from the integrative analysis in a hypertension study

Name	Chromosome	Starting location	Ending location	# of SNVs	*p*-value
*UBAC1*	9	138823836	138854205	287	9.86×10^−5^
*MEGF11*	15	66186838	66546725	2989	2.14×10^−4^
*IFI44L*	1	79085201	79112428	207	5.35×10^−4^
*MFGE8*	15	89440944	89457653	161	1.27×10^−3^
*ANKDD1A*	15	65203490	65251983	464	1.29×10^−3^
*PDZD2*	5	31798110	32111928	3655	1.96×10^−3^
*TBX4*	17	59532864	59561970	221	2.15×10^−3^
*IGSF3*	1	117116060	117211147	429	2.42×10^−3^
*TMEM61*	1	55445562	55458886	170	3.90×10^−3^
*FAM46B*	1	27330739	27340321	63	7.29×10^−3^

## Conclusion

To facilitate the integrative analysis of omic data, we proposed a unified non-parametric method to detect the joint association of multi-level omic data with various types of phenotypes. There are three main contributions of the proposed IU method. First, it provides robust performance for various types of phenotypes, including binary, Gaussian and heavy-tailed distributions, due to the robustness of U statistics. Second, the proposed integrative U test achieves higher or comparable power compared to existing methods (e.g., VCT) under different types of interaction models. Finally, we also provide a simple sample size/power calculation to facilitate the design of multi-level omic studies.

The connection between the proposed method and variance component tests is that all test statistics are in the form of kernel quadratic framework as seen in “Simulation” section. It also connects to several other U-statistic-based methods [10, 27]. As a similarity-based test, the IU method is proposed as a non-degenerated U statistic, which follows a normal distribution. One advantage of using a non-degenerated U statistic is the computational accuracy with no distribution approximation. If we centralize the phenotype, it becomes a degenerated U test, which follows a mixture chi-square distribution.

The IU test can be extended to handle more than 2 levels of omic data. For instance, a modified IU test can be applied for 3 levels of omic data. Besides the SNVs and gene expression, we further introduce *R*_*i*_ as the DNA methylation for subject *i*, and use kernel matrix *K*_3_(·,·) to measure the DNA methylation similarities. The IU test statistic can then be defined as

$$\begin{array}{@{}rcl@{}} U_{n} = \frac{1}{n(n-1)} \sum_{i \neq j} K_{1}(Y_{i},Y_{j}) K_{2}(S_{i},S_{j}) K_{3}(R_{i},R_{j}) \int G_{i}(t) G_{j}(t) dt. \end{array} $$

The choice of *K*_3_ is similar to *K*_1_ and *K*_2_ as discussed in “Methods” section. Following the same argument for Theorem 1, we can show that this modified IU test also follows an asymptotically normal distribution. In addition, with multiple genes (e.g., genes in a biological pathway) and the corresponding gene expression levels, the gene expression level S can also be modeled as a function. For such purpose, the similarity measure *K*_2_(*S*_*i*_,*S*_*j*_) can be modified as $\tilde K_{2}(S_{i}(t),S_{j}(t))$ where $\tilde K_{2}(\cdot,\cdot)$ measures the similarity between two functions. The asymptotic property can be derived based on the same argument for Theorem 1.

One potential limitation of this study is that gene expression is assumed to be independent of SNVs. One technical reason of making such assumption is that, under the stochastic process setup, it is hard to model the association between the gene expression variable *S* and the underlying stochastic process *SP*(*η*(*t*),*Γ*(*s*,*t*)). Finding an appropriate way to model correlations among omic data is a challenging topic that is worth of further investigation. Nevertheless, if real data indicates correlations among different levels of omic data, one way to overcome this issue is to adopt methods introduced by [27] and [28].

## Additional file

Additional file 1 The proof of Theorem 2.1 can be found in the Appendix. (PDF 94 kb)

## Abbreviations

Adj-SKAT: Adjusted sequence kernel association test
DE: Double exponential
HTN: Hypertension
IU: Integrative U test
SAFDGS: San Antonio Family Diabetes/Gallbladder Study
SAFHS: San Antonio Family Heart Study
SKAT: Sequence kernel association test
VCT: Variance component test
WGS: Whole-genome sequencing
