# Supplementary material for: An integrative U method for joint analysis of multi-level omic data
Source: BMC Genet. 2019 Apr 10;20:40. doi: 10.1186/s12863-019-0742-z (PMC6457037; doi:10.1186/s12863-019-0742-z)
Supplement: Supplementary file 1 — The proof of Theorem 2.1 can be found in the Appendix. (PDF 94 kb) [file 12863_2019_742_MOESM1_ESM.pdf]

Supplement to

"An integrative U method for joint analysis of multi-level omic data"

by Pei Geng, Xiaoran Tong and Qing Lu

1 Appendix

Proof of Theorem 2.1:

By the central limit theorem for stochastic processes, we have

$$\sqrt{n}(\bar{G}(t)-\eta(t)) \to GP(0,\Gamma(s,t)).$$

In other words, $\bar{G}(t) \sim GP(\eta(t),\Gamma(s,t)/n)$ given a sufficiently large n. By using the property of Gaussian processes, we further obtain

$$E(||\bar{G}||^2)=||\eta||^2+tr(\Gamma/n) \text{ and } Var(||\bar{G}||^2)=2tr\Big(\big(\frac{\Gamma}{n}\big)^{\otimes 2}\Big)+4\sum_{k=1}^{m}\lambda_k\delta_k^2/n.$$

Based on the moments of sample means, $E\bar{Y}^4=\mu_Y^4+6\mu_Y^2\sigma_Y^2/n+o(1/n)$ and $E\bar{S}^4=\mu_S^4+6\mu_S^2\sigma_S^2/n+o(1/n)$, and omitting the high order of $1/n$, we can obtain the asymptotic variance of $\hat{\mu}_0$,

$$\begin{aligned} Var(\hat{\mu}_0) &= E\bar{Y}^4E\bar{S}^4E||\bar{G}||^4-\{E\bar{Y}^2E\bar{S}^2E||\bar{G}||^2\}^2 \\ &= 4\{\mu_Y^4\mu_S^4\sum\lambda_k\delta_k^2+\mu_Y^2\sigma_Y^2\mu_S^4+\mu_S^2\sigma_S^2\mu_Y^4\}||\eta||^4/n+o(1/n) \\ &\equiv 4\sigma_2^2/n+o(1/n). \end{aligned}$$

Therefore, we obtain

$$\sqrt{n}(\hat{\mu}_0-\mu_0)\to N(0,4\sigma_2^2).$$

Combining the asymptotic normality in (2.1) of Section 2.3, we eventually have

$$\sqrt{n}(U_n-\hat{\mu}_0)\to N(0,\sigma^2),$$

where

$$\sigma^2 = 4(\sigma_1^2-\sigma_2^2)=4\sum\lambda_k\delta_k^2\{\mu_Y^2\mu_S^2(\mu_Y^2\sigma_S^2+\mu_S^2\sigma_Y^2+\sigma_Y^2\sigma_S^2)\}+4\mu_Y^2\mu_S^2||\eta(t)||^4\sigma_Y^2\sigma_S^2.$$

A consistent estimator of σ^2 is obtained as

$$\hat{\sigma}^2 = 4\sum \hat{\lambda}_k \hat{\delta}_k^2 \{\bar{Y}^2 \bar{S}^2 (\bar{Y}^2 s_S^2 + \bar{S}^2 s_Y^2 + s_Y^2 s_S^2)\} + 4\bar{Y}^2 \bar{S}^2 ||\bar{G}||^4 s_Y^2 s_S^2,$$

where s_Y^2 and s_S^2 are the sample variances of Y and S. $\hat{\lambda}_k$ and $\hat{\phi}_k$ are respectively the eigenvalues and eigenfunctions of $\hat{\Gamma}(s,t) = \frac{1}{n}\sum_{i=1}^{n}(G_i(t) - \bar{G}(t))(G_i(s) - \bar{G}(s))$, and $\hat{\delta}_k = \int_0^1 \hat{\phi}_k(t)\bar{G}(t)dt$.
